# Efficiency Analysis of Syrian Refugees’ Healthcare Services in Turkey and Other 3RP States

**DOI:** 10.3390/ijerph192214986

**Published:** 2022-11-14

**Authors:** Emmanuel Achiri, Mustapha D. Ibrahim

**Affiliations:** 1 Department of Political Science and International Relations, Faculty of Business and Economics, Eastern Mediterranean University, Famagusta 99628, Turkey; 2 Industrial Engineering Technology, Higher Colleges of Technology, Sharjah P.O. Box 7947, United Arab Emirates

**Keywords:** efficiency, data envelopment analysis, normative analysis, refugee healthcare

## Abstract

Access to healthcare for refugees is often contextually and conceptually diverse. This study set out to evaluate the efficiency of the healthcare services provided for Syrian refugees in Turkey and other refugee response and resilience plan (3RP) states. Data envelopment analysis is utilized for efficiency analysis using primary healthcare system indicators. Efficiency is broken down into pure technical efficiency and scale efficiency to identify causes of inefficiency. Normative analysis is used to employ a teleological approach to better understand current refugee healthcare policies. The findings show a decline in the overall efficiency for Turkey and other 3RP states. However, Turkey’s operational practices could be a model for other 3RP states due to its relatively high pure technical efficiency. Scale inefficiency negatively impacts the overall efficiency of Turkey’s service, while other 3RP states exhibit a rather consistent performance. The study concludes that Turkey’s healthcare system for refugees is inefficient, resulting in inadequate access for Syrian refugees. As such, operational and scale efficiency must be synergized for Turkey to fulfill its obligation to provide adequate healthcare for Syrian refugees. Additionally, COVID-19 was found to have exacerbated the challenges Syrian refugees face accessing healthcare. Policy recommendations have been made in line with the findings of the study.

## 1. Introduction

The war in Syria continues to drive one of the largest refugee crises in the world. Syria’s closest neighbors, Turkey, Lebanon, Jordan, Egypt and Iraq disproportionately host approximately 5,662,097 Syrian refugees [1]. Turkey alone hosts about 3.7 million of these Syrian refugees under temporary protection—the world’s largest refugee population—and an additional 320,000 asylum seekers and refugees under international protection [2]. While the number of Syrian arrivals in Turkey has been reducing as Turkey has begun to restrict access to its borders, sometimes violently, and ended visa-free entry for Syrians, many fleeing the war continue to make their way to Turkey in hope of protection [3]. These refugees have special needs, including the need to access healthcare, education, the labor market, housing and other basic necessities, that often strain the economy of the host state. With Europe making it harder for Syrian refugees to access international protection, regional states have had to shoulder this enormous burden [4]. In the case of Turkey, the number of Syrian refugees has put a strain on the country’s capacity [5].

Recognizing the need to support countries hosting large numbers of Syrian refugees, the Regional Refugee and Resilience Plan (3RP) was created in 2015 to help refugees in these countries and systematically support host communities [6]. The 3RP is a two-part regional plan covering five standalone country chapters: Turkey, Jordan, Lebanon, Egypt and Iraq. The first part addresses the humanitarian and protection needs of refugees, and the second addresses the resilience, development and stabilization needs of impacted communities. By combining the response plans of Turkey, Lebanon, Jordan, Iraq and Egypt in a coordinated network, 3RP is simultaneously country-driven and regionally coherent with a goal to ensure that Syrian refugees live a life in dignity in their host countries including access to adequate healthcare.

The right to the highest attainable standard of health is essential to a dignified life for many Syrian refugees. Improvements in the health services available to refugees can only be made by evaluating previous performance and benchmarking relatively better practices. The intention here is to investigate empirically whether 3RP states, with an emphasis on Turkey, utilize their limited resources in an efficient manner such that it guarantees the basic health needs of Syrian refugees.

Turkey is chosen as the focal point of analysis because of the significant proportion of Syrian refugees it hosts, that is, the world’s largest Syrian refugee population. It is also the primary destination for Syrian refugees and has received significant financial support from the European Union and the World Bank under the Syrians Under Temporary Protection (SUTP) program to support the humanitarian and developmental challenges for refugee and host communities [7].

Turkey’s response has been the subject of many studies focusing on the status of protection for Syrian refugees; how Turkey interprets its obligations towards refugees, particularly temporary protection [8]; the experiences, integration and attitude of Syrian refugees in Turkey [9]; the reception policies and practices of state and non-state actors in Turkey [10], integration practices, policies and responses to refugee immigration [11]; the impact of culture, religion, belief systems and political discourse on refugees’ healthcare access [12]; and the quality of healthcare access for Syrian refugees [13,14].

The importance of this study compared to other studies on Syrian refugees in Turkey lies in its mixed-methods approach and its specificity. Unlike other studies that simply evaluated the access to and quality of healthcare for Syrian refugees in Turkey, this study goes a step further to assess the efficiency of Turkey’s resource utilization to understand whether Turkey is using its limited resources efficiently to provide for the basic health needs of Syrian refugees. By extension, it also seeks to ascertain whether there is a correlation between (in)efficient use of resources and the access to and the quality of healthcare Syrian refugees are entitled to. Building on the work of Kuriki and Ikeda [15], which used the data envelopment analysis (DEA) model to evaluate the efficiency of the Syrian refugee healthcare system in Turkey, Lebanon, Jordan, Iraq and Egypt between 2015 and 2018, this study extends the period of study to 2020. Furthermore, this study, unlike Kuriki’s that focused solely on operational efficiency, breaks down the efficiency of the healthcare systems into operational and scale efficiency for a more in-depth analysis and understanding of their performance. Additionally, a legal normative understanding of the meaning of access to healthcare is used to compliment the DEA analysis, assess policy impact and prescribe appropriate policy recommendations. It contributes to the existing literature in four ways by: assessing the efficiency of Turkey’s healthcare resource utilization by identifying and contextualizing the operational and scale (in)efficiency of providers; benchmarking best practices in both operational and scale efficiency for performance improvement; evaluating the link between resource utilization efficiency and the quality of healthcare access for Syrian refugees; and providing practical and implementable policy recommendations.

### 1.1. The Right to Healthcare

The right to health is an inclusive right in that a wide range of factors are needed to enable us to live a healthy life: access to safe food, drinking water, clean sanitation, adequate housing and nutrition, gender equality, access to health-related information and education, and healthy working and environmental conditions [6]. It also contains certain entitlements: access to essential medicine, the right to prevention and treatment and the right to a health system that provides quality of opportunity for everyone to “enjoy the highest attainable level of health”, among others [16]. The right to health is considered the cornerstone of strong and effective health systems and essential to the growing body of human rights, and an equitable and healthy society. The right to health, as with other human rights, is particularly concerned with disadvantaged people [17].

Human rights are interdependent and interrelated; the enjoyment of one right directly depends on the realization of another. Specifically, the right to health depends on the realization and enjoyment of the rights to information, privacy, a life of dignity, housing, work, education, movement, freedom from torture and numerous others. Several international human rights instruments contain provisions relating to the right to health. Article 25 (1) of the Universal Declaration of Human Rights states the right of everyone “to a standard of living adequate for himself and of his family” [18]. Similarly, Article 5 of the International Convention on the Elimination of all Forms of Racial Discrimination [19], Articles 11 and 12 of the Convention on the Elimination of All Forms of Discrimination against Women [20], Article 24 of the Convention on the Rights of the Child [21], and Article 16 of the African Charter on Human and Peoples’ Rights [22] among others, also confer this right. Notwithstanding these, the key international human rights instrument that elucidates the right to health is the International Convention on Economic, Social and Cultural rights (ICESCR) [23].

Article 12 of the ICESCR defines health and specifies the obligations of states vis-a-vis the realization of “the right of everyone to the enjoyment of the highest attainable standard of physical and mental health” [24]. It is noteworthy that the drafters of the Convention intended to define health differently from the preamble of the World Health Organization’s constitution: “a state of complete physical, mental and social well-being and not merely the absence of disease or infirmity” [25]. The WHO’s definition had long been criticized, with some claiming that promoting this definition would lead to “absurd claims to outlaw diseases, infirmities caused by ageing, and even mortality” [26]. Drafting history shows that the ICESCR meant for the right to health to also include other socio-economic factors essential for its enjoyment, including access to food, portable water, clean sanitation, adequate housing, and safe and healthy working conditions [26].

However, the definition stated in Article 12 (1) should not be interpreted as the right to be healthy, which could be affected by a number of other factors such as biology, socio-economic preconditions and the available resources of a state. Instead, it is meant to be understood as the right to enjoy the services, facilities and goods necessary for the realization of good health. It is for this reason that it is more accurately described as the right to the highest attainable standard of physical and mental health [25], meaning that an individual’s level of health will be determined by elements unique to that individual and state resources [27], or, simply put, what society does to guarantee that people can live healthy lives [28]. Therefore, the state’s duty lies in providing the necessary conditions, resources, and goods and services to allow individuals to enjoy the highest attainable standard of health, and not to ensure that everyone is healthy.

As such, one could say that the right to health includes the following essential and interconnected components, although specific applications depend on the prevailing conditions within states party to the Convention: (i) a functioning healthcare system with sufficient facilities, and goods and services, depending on the developmental level of the state; (ii) health facilities available to everyone without discrimination, within a safe distance (physical accessibility), affordable (economic accessibility) and the right to seek and receive information (information accessibility); (iii) culturally, medically and scientifically appropriate healthcare in line with standard practices; and (iv) overall quality healthcare [26].

Non-discrimination is a key element of the right to health. Accordingly, health services, goods and facilities must be made accessible and available to everyone, and of good quality, particularly for the most vulnerable groups in society [26]. Therefore, states have a special obligation to ensure that those who do not have sufficient means can access available health facilities per the core obligations of states, which require states to at least provide essential facilities guaranteeing each right in the ICESCR, including primary healthcare to ensure that access to health facilities, services and goods are available without discrimination.

International human rights law is realistic and the ICESCR recognizes that the right to and enjoyment of the highest standard of health is subject to resource availability and progressive realization [29]. Essentially, the requirement is for states to “take steps” towards realizing this right, which the International Law Commission interprets as taking immediate steps within a reasonably short time (progressive realization) [24]. These steps include legislative measures, judicial remedies, administrative measures, and educational, financial and social measures that guarantee the highest attainable standard of health [30]. The right to health also requires indicators and benchmarks to monitor progressive realization [29]. However, this is often complicated because it requires access to comparable statistical data from different periods in order to evaluate trends, which many states simply lack [31]. In the case of Syrian refugees in Turkey and other 3RP states, the availability of data allows for the benchmarking and the possibility to monitor and evaluate the quality of access to the healthcare available to them.

### 1.2. Access to Healthcare for Refugees

Access to healthcare for refugees is often contextually and conceptually diverse, and measuring whether this access is adequate or not is always an arduous task. Other scholars have also sought to investigate the access refugees have to adequate healthcare. Lebano et al. focused on the impact that refugees and other migrant groups have had on the European healthcare system arguing that refugees in Europe tend to have higher prevalence of mental distress than non-refugees [32]. Some have sought to understand the relationship between trauma symptoms and barriers to healthcare among Southeast Asian refugees in Connecticut [33], and realized access to healthcare—the link between access to and use of healthcare services by refugees in Germany [34]. Four dimensions have also been identified as affecting access to healthcare for refugees, culture, stigmatization, discrimination and language [35], while yet another concluded that culture, language and difficulties in navigating social and health systems in host countries are some of the barriers to refugees’ access to healthcare [36]. In Jordan, the health challenges of Syrian refugees have become a political and socio-economic crisis [37]. In Lebanon and Jordan, communicable diseases were the most acute medical problems for refugees, mostly affecting Syrian refugee children [38]. Studies in some low-income countries reveal that refugees living in urban areas have fairly good access to primary healthcare services, although secondary and tertiary healthcare remains problematic [39].

This study sets out to evaluate the efficiency of Syrian refugees’ access to healthcare in 3RP states, in particular in Turkey, using DEA analysis. The purpose is not simply to use efficiency scores to assess the effectiveness of healthcare delivery, but to measure efficiency understood as adequate healthcare services provided to Syrian refugees given the available resources.

### 1.3. Legal Framework for Healthcare Access for Syrian Refugees in Turkey

The Turkish interpretation of the 1951 Refugee Convention is based on geographical limitations and Turkey only recognizes refugees originating from Europe. However, following global criticism amid the influx of Syrian asylum seekers into Turkey, as well as the fact that Turkey has ratified and is bound by international human rights instruments such as the Covenant on Economic, Social and Cultural Rights, which places obligations on signatory states to take immediate and appropriate steps to guarantee the rights therein for all within their jurisdictions without discrimination, Turkish authorities adopted the Law on Foreigners and International protection (LFIP) in 2013, inspired by European Union law [40]. The LFIP reaffirmed Turkey’s international protection obligations and established a legal framework for asylum. It also led to the creation of the Directorate General of Migration Management (DGMM), which has since taken over all tasks relating to international protection within Turkey in 2018 [41]. In 2014, the Council of Ministers issued Turkey’s most significant legal response—the Temporary Protection Regulation (TPR). Under the TPR, Syrian refugees were given access to certain temporary protection benefits such as access to healthcare services, to the labor market, and to education [42]. The TPR was intended to “determine the procedures and principles pertaining to temporary protection proceedings that may be provided to foreigners, who were forced to leave their countries”. The TPR vested the power to provide temporary protection with the Council of Ministers (Article 9), provided for emergency health services to be offered to refugees who have been determined to have emergency health needs (Article 20), put the power of registration to be carried out by Directorate General personnel (Article 21), made provision for temporary protection ID cards to be given by Governorates (Article 22), and for referral to accommodation centers with priorities given to persons with special needs (Article 23), provided access to health, education, the labor market and social assistance (Article 26), specified the types of health services offered (Article 27), and established AFAD (Disaster and Emergency Management Presidency) to operate and manage temporary accommodations (Article 37).

**Figure 1:**
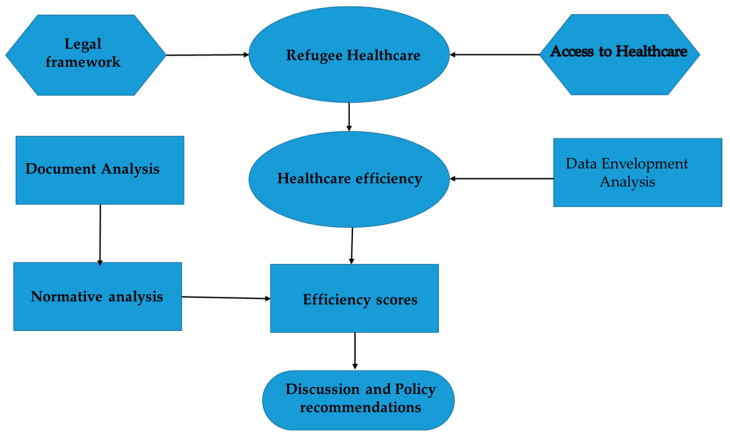
Analysis framework.

Figure 1 presents the study framework employed for a holistic analysis of refugee healthcare from quantitative and qualitative viewpoints.

## 2. Methodology

This study adopts a mixed-methods approach, utilizing both qualitative and quantitative research methods to evaluate the efficiency of healthcare provision for Syrian refugees in the 3RP countries and Turkey in particular. Mixed-methods research brings insights from different research methods in the analysis of a phenomenon [43]. The mixed-method data analysis used in this study provides a richer, more evaluative understanding of the type and quality of the healthcare access Syrian refugees are entitled to in Turkey. It builds on the previous work by Kuriki and Ikeda using data envelopment analysis and extends this further by evaluating overall efficiency, and breaking it downing into operational efficiency and scale efficiency from 2015 to 2020. Decomposing the DEA efficiency scores allows factors affecting performance to be investigated. Document analysis of primary and secondary sources is also conducted, in addition to a legal positivist and normative research analysis.

### 2.1. Data Envelopment Analysis

DEA is a non-parametric method for evaluating the relative efficiency of systems, known as decision-making units (DMUs). DEA was introduced by Charnes, Cooper and Rhodes (CCR) in 1978 [44] with the CCR model and modified by Banker, Charnes and Cooper in 1984 [45] with the BCC model. DEA is a widely-used technique for evaluating complex systems such as healthcare [46] and environmental sustainability [47]. Its ability to analyse multiple inputs and outputs simultaneously makes it the preferred method for empirical analysis with policy implications. To assess efficiency, DEA uses linear programming to project a frontier of efficient units that envelop the inefficient units. The approach assesses efficiency by examining different input–output combinations. The DMU that utilizes the least inputs in achieving the most outputs is identified as efficient relative to other DMUs. This provides valuable information on performance measurement by benchmarking systems that transform the same resources to efficient outputs. It also provides insights into causes of inefficiency and identifies improvement strategies.

DEA is widely applied in healthcare assessment, including hospitals [48] and health directives [49]. The choice of model typically corresponds to the researcher’s definition of efficiency [15]. The input orientation model measures the extent to which a unit optimizes inputs to attain outputs, while output orientation measures the optimal outputs with existing inputs. If a unit is inefficient under input orientation, then the recommendation is made to reduce inputs while producing the same outputs. On the other hand, an inefficient unit in output orientation is recommended to increase outputs with the same number of inputs [50]. The directional model introduced by Chambers et al. [51] offers a confluence of both models and is utilized in this study. The directional model nests the input and output-oriented models, avoiding the drawbacks of each.

In DEA, the constant return to scale (CRS) efficiency or overall efficiency includes scale efficiency (SE) and pure technical efficiency (PTE). The variable return scale (VRS) efficiency represents the PTE. In the directional distance model applied in this study, SE=CRS Eff.−VRS Eff. [52]. To obtain efficiency scores, a unit (period) with inputs and outputs $\left(x_{0},y_{0}\right)$ is projected in a preassigned direction; $g=\left(-g_{x}^{-},g_{y}^{+}\right)\neq 0_{m+s},g_{x}^{-}\in {ℜ}^{m}\;and\;g_{y}^{-}\in {ℜ}^{s}$ in a proportion β. The linear program to obtain efficiency scores is:

(1) $$\begin{array}{l} \underset{\beta ,\lambda }{\max}\;\;\beta \\ subject\;to\\ \;\;\;X\lambda \leq x_{0}-\beta g_{x}^{-}\\ \;\;\;X\lambda \geq y_{0}+\beta g_{y}^{-}\\ \;\;\;\lambda \geq 0 \end{array}$$

Breaking down overall efficiency into PTE and SE helps identify the cause of inefficiency [53]. PTE is associated with the operational level of the DMU. It represents the operational aspects of efficiency. SE indicates the optimal size utilization of the DMU and how well the DMU is utilizing the size of their operations. DMUs that are scale inefficient need to identify the optimal scale required for efficient performance. In this study, we evaluate overall efficiency, PTE and SE of refugee healthcare services.

The data analysed are obtained from reports produced by the Syrian 3RP scheme [54], as well as other reports from the “EU Regional Trust Fund in Response to the Syrian Crisis” and the “EU Facility for Refugees in Turkey” provided by the European Commission [55].

### 2.2. Document Analysis

Document analysis is a systematic procedure for reviewing documents by analyzing and interpreting data to gain new understanding and knowledge [56]. It is a process involving the thorough examination of primary and secondary sources of data to identify pertinent information by recognizing patterns and thematic analysis [57]. Although document analysis also involves the review of primary and secondary sources of literature, it should not be confused with a literature review. The former is a form of analyzing data indirectly, and the latter a simple overview of research studies [58]. Document analysis is a process by which documents are evaluated in order to develop understanding and empirical knowledge. Journals, books, manuals, maps, newspapers, press releases, etc., are examples of such documents that can be analyzed [59]. It provides the researcher with a more accurate understanding of the socio-cultural, political and economic contexts within which ideas are conceived [28].

Atlas.ti was used to support the analysis of relevant primary and secondary sources. The criteria for selecting the documents to be analyzed was based on background information, intended audience, political purpose and the purported agenda of the relevant document. For this particular study, analyses of primary sources: official documents by Turkish authorities, international Covenants and treaties, 3RP reports, commentaries by the international law commission, publications by inter-governmental organizations (IGOs) and non-governmental organizations (NGOs), and Turkey country reports, which included published interviews with refugees conducted by prominent researchers, were conducted [30]. Some secondary sources were also analyzed as part of this process. The process was as follows: relevant excerpts based on interesting features were initially highlighted during first readings and, thereafter, codes (access to quality healthcare, health as an inclusive right, health as a fundamental right, service provision efficiency, health as a state of complete physical, mental and social well-being, factors affecting access to healthcare, healthcare standards, burdens on healthcare systems, among others) were generated and assigned to these excerpts. These codes were subsequently sorted to search for two common themes; (a) the right to health as an inclusive right and (b) all services, goods and facilities must be available, accessible, acceptable and of good quality. The connection between these two themes was assessed as being fundamental to answering the question; “what is the right to health?” Understanding the meaning of the right to health and what adequate access to healthcare means are essential components of this study. That is, in order to ascertain whether there is a correlation between (in)efficient use of resources and access to and the quality of healthcare available to Syrian refugees, we need to first understand what the right to health means.

### 2.3. Legal Normativism

An important part of this study involves understanding what the right to health, or better described, how the right to the highest attainable standard of health, is to be understood. Normative legal theory is evaluative and seeks to explain the law as it ought to be (normative cognition). As such, when interpreting the law, it is also important to take into consideration the travaux preparatoires and intentions of the lawmaker (teleological cognition) [60]. A key feature of normative analysis is its methodological and critical nature. This study utilizes a normative methodology to investigate the intentions behind the formulation of the definition of the right to health under international human rights law; in particular, the provisions of the Covenant on Economic, Social and Cultural Rights and the obligations it imposes on states. To do this, the researchers adopted a teleological approach, seeking to understand the intention of the lawmaker. The primary document of analysis for this purpose was the ICESCR Convention, which is the authoritative document elucidating the right to health. Attempting to understand the intention of the lawmaker is often an arduous task as there could be many different interpretations. As such, part of this normative analysis included investigating the interpretations of the Committee on Economic, Social and Cultural Rights (CESCR) [30], which was established by the Economic and Social Council (ECOSOC) to monitor state party adherence to the ICESCR. Additionally, commentaries by the international law commission and the OHCHR [16] were also read as part of this process of ascertaining the meaning of the right to health. By contextualizing the meaning of the right to health, the study was able to determine whether Turkey’s use of resources translated to quality healthcare for Syrian refugees. Without a normative understanding of what the right to health is, it would be impossible to evaluate whether Syrian refugees benefit from quality access to healthcare or not, and whether this satisfies the intention of the drafters of the ICESCR.

## 3. Results and Discussion

### 3.1. Healthcare Indicators and Efficiency Results

The study utilizes the primary indicators of healthcare system efficiency as presented and discussed in the literature [46,61,62]. In evaluating healthcare system efficiency, the input indicators are categorized into economic/financial resources and human resources [63]. Studies such as Ibrahim et al. [53] and Önen and Sayın [64] used healthcare expenditure and trained personnel as input indicators. Similarly, in the analysis of refugee healthcare by Kuriki and Ikeda [15] “trained health personnel” and “health expenditure” are used as input variables. The “number of trained health personnel” is a necessary input when evaluating access to health because health workers are the cornerstone of health systems [65], while “health expenditure” can result in better healthcare provision and is a good gauge for an assessment of the final consumption of healthcare services [66]. The output indicator is defined according to the context of the analysis [67]. The output indicator should answer the question: how well are the inputs utilized? Similarly, in the refugee healthcare services analysis of Kuriki and Ikeda [15], “the number of primary healthcare consultations” is used as the output indicator. The number of primary healthcare consultation is a good metric for evaluating access to health services [68].

Table 1 presents the descriptive statistics of the variables used in the efficiency analysis. The average number of trained personnel continues to increase across the evaluated period. Similar observations are made in the expenditure and health consultations.

**Figure 2:**
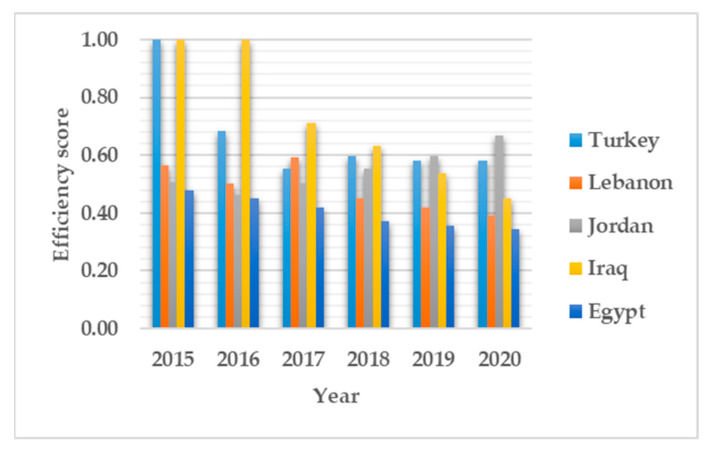
Overall efficiency scores.

As per the DEA efficiency analysis and model (1), an efficiency score of one indicates a relatively efficient performance in that period, and less than one signifies an inefficient performance. The refugee healthcare efficiency in this context represents the adequate healthcare services provided for refugees given the available resources of trained personnel and health expenditure. Table 2 presents the descriptive statistics of the efficiency for the evaluated period. The result shows absence of uniformity in refugee healthcare provision in Turkey and other 3RP states. The overall efficiency score is a combination of both operational and capacity utilization of the healthcare system. The average overall efficiency continues to decline over the evaluated period. Results for all 3RP states show a 13% decrease in overall efficiency between 2015 and 2020. This indicates a gross underperformance of refugee healthcare services over time. Figure 2 illustrates the detailed overall efficiency by each country. In 2015, Turkey and Iraq performed efficiently. Only Iraq performed efficiently in 2016, and Egypt was consistently the least efficient country overall. The performance of Lebanon and Jordan was relatively consistent, though inefficient. In 2020, Jordan had an efficiency score of 0.67, the maximum in that period. Though the standard deviation continuous to decrease overtime, the overall inefficiency is alarming.

**Figure 3:**
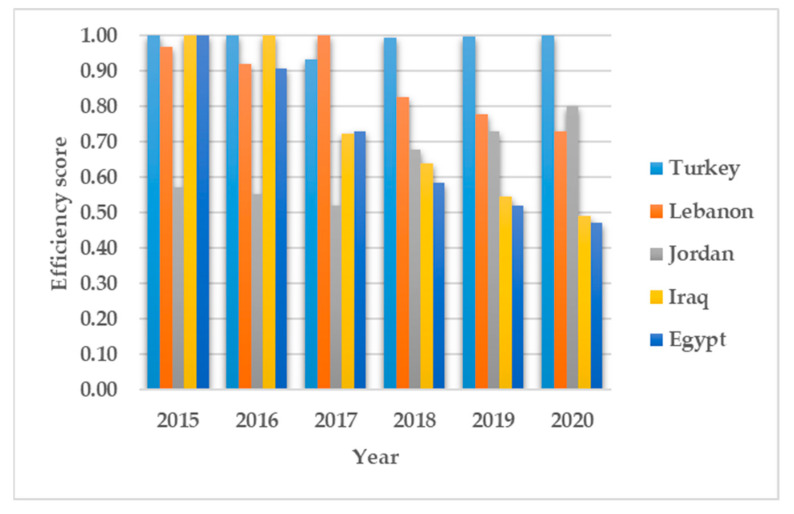
Pure technical efficiency scores.

**Figure 4:**
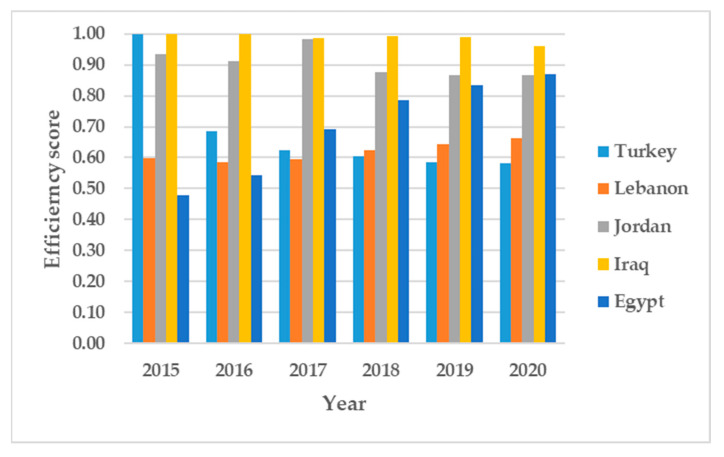
Scale efficiency scores.

The cause of inefficiency might be unique for each country. The efficiency decomposition into pure technical efficiency and scale efficiency reveals the cause of inefficiency for each country in each period. Table 2 shows a relative decline in operational performance represented by pure technical efficiency. The average pure technical efficiency ranges between 0.71 and 0.91. In 2019 and 2020, Turkey appears to have an efficient operational refugee healthcare system. Other 3RP states performed inefficiently too. The detailed pure technical efficiency in Figure 3 shows minimal decline in the operational performance of refugee healthcare systems. Lebanon also shows a relatively high operational efficiency prior to 2018. Jordan shows hints of operational improvement in 2020 compared to other periods. Lebanon, Jordan, Iraq and Egypt show average operational efficiencies of 0.87, 0.64, 0.73 and 0.70, respectively, compared to Turkey’s 0.99. Turkey’s high operational efficiency across the evaluated period signifies that lessons can be learned by other 3RP states from Turkey’s operational practices. The scale efficiency (Figure 4) that represents the optimal use of existing capacity shows Iraq to be most efficient. Turkey on the other hand, shows a high scale inefficiency score with an average of 0.68 in 2020 when compared to its 2015 SE score. Iraq and Jordan attained high SE efficiency scores of 0.99 and 0.91, respectively.

The results show Turkey to be the benchmark for operational efficiency. By comparing Turkey’s efficiency to that of other 3RP states that also host large numbers of Syrian refugees, it is possible to see that Turkey’s healthcare system for refugees is overall inefficient due to low scale efficiency (SE), even though it has an outstanding operational efficiency. Components of operational efficiency include: infrastructure, care delivery standards, regulatory compliance safeguards, and performance trackers and matrices [69]. The results are consistent with the findings of Kuriki and Ikeda, which highlight Turkey’s deliberate actions towards improving the effectiveness and quality of its trained medical personnel. From Figure 2, Figure 3 and Figure 4, Lebanon, Jordan and Egypt appear to have the most consistent performance on all of the efficiency dimensions, even though they are inefficient. Turkey, on its part, exhibits a rather inconsistent performance on overall and scale efficiency. This could be attributed to the high number of Syrian refugees settling in Turkey [2], as well as the limitations of the temporary protection regulation system [70]. Based on the overall efficiency scores and efficiency decomposition, it can be deduced that scale inefficiency is the primary cause of refugee healthcare inefficiency in Turkey. Therefore, to improve the efficiency of healthcare for Syrian refugees, Turkey needs to scale up services while maintaining its operational efficiency. Given the limited resources available, it is imperative to implement the strategic deployment of services to enhance scale efficiency that could translate to better refugee healthcare services. 

### 3.2. Efficiency and Normative Analysis Results

As the DEA analysis from 2015 to 2020 shows, despite being operationally efficient, over the years, Turkey’s overall efficiency has plummeted as a result of declining scale efficiency. Meaning that, while performance and care delivery standards, regulatory compliance safeguards and performance trackers and metrics have been adequate, the scale of services to Syrian refugees has not been sufficient, causing the health delivery system to not be very efficient. We argue that an inefficient healthcare system, as is the case with the Turkish healthcare system for Syrian refugees, demonstrated by the DEA analysis, is incapable of providing adequate healthcare. A key component of the right to health understood normatively as the right to an adequate standard of healthcare is the existence of a functioning healthcare system with sufficient facilities, and goods and services [60]. The question is whether Turkey’s healthcare system for Syrian refugees meets this criterion. The answer is simple. The number of trained medical personnel is insufficient (scale inefficiency), in addition to other quantity-related service problems. The DEA analysis shows us that SE impacts overall efficiency (Figure 2 and Figure 4), leading to an overall inefficient healthcare service system. As such, one cannot say that the criterion for an adequate standard of healthcare has been met. Scale inefficiency is also affected by the high number of Syrian refugees making their way to Turkey, which has overburdened the healthcare system. That is, as the numbers of Syrian refugees in Turkey have continued to increase and the scale of trained health personnel has not meet these new numbers, the quality has deteriorated.

These findings are consistent with the literature. Turkey’s operational efficiency is good because of the care delivery standards of its trained medical personnel. One reason for this is because Turkey has integrated some Syrian health professionals within its healthcare system, resulting in many health centers in rural areas being staffed by both Syrian and Turkish health professionals, thereby reducing language barriers, increasing accessibility and improving the quality of healthcare for many refugees who have a preference to consult with Syrian doctors [71]. However, in the metropolitan cities such as Istanbul where many Syrian refugees move to in search of work and better living conditions, refugees often face difficulties finding health facilities staffed with medical professionals who can communicate in Arabic [71], pointing to a problem of scale. As a result, refugees who would normally benefit from free access to healthcare facilities have turned to out-care in refugee-run clinics, reinforcing the need to increase scale by adding more trained Turkish medical professionals or by integrating more Syrian medical professionals into the Turkish healthcare system [65]. Furthermore, treatment in migrant health centers (MHC) is often rushed and cursory because the few medical professionals are overburdened in overcrowded hospitals [71], worsened by health providers having to double as “gate keepers” for refugees “on the move” [68]. Because the number of trained staff is not sufficient, this means health professionals have to work long hours leading to burnouts, which in turn affects the quality of the healthcare they are able to provide [72].

Although scale inefficiency is the main cause of overall inefficiency, it does not mean that the problem ends there. In fact, this study contends that once a healthcare system’s efficiency is affected by one or more factors, this by extension affects the entire system, making it inefficient (Figure 2), which reflects in many other ways. For instance, the development of an informal healthcare sector run by Syrian doctors and NGOs has allowed the Turkish state to relegate the needs of refugees to this sometimes expensive informal and precarious private realm. This has only served to shut many refugees out of the health system [65]. Additionally, there are structural problems inherent within the temporary protection system for Syrian refugees that negatively impacts their access to quality healthcare. For instance, the obtention of the TPR ID card, which offers Syrian refugees rights including access to health services, is often tedious. Without this card, refugees are unable to access healthcare [9]. Furthermore, the rise in the number of Syrian refugees in Turkey has created backlogs and overburdened an already fragile infrastructure [14], and by 2018, this had worsened as Turkey adopted more restrictive regulatory practices making it harder for refugees to register under the TPR [13]. Without this registration, they cannot access health facilities run by the government. Moreover, obtaining a temporary protection card did not necessarily translate to easier access to healthcare for some refugees as accessibility and taking appointments at healthcare facilities remained problematic [13]. Additionally, the emphasis on the temporary nature of the 187 migrant health centers (MHCs) has contributed to uncertainty and anxiousness among refugees’ increasing mental stress [9]. Even when refugees accessed health facilities, pharmacies sometimes did not provide prescriptions because securing reimbursements from the state was often difficult [13].

The outbreak of COVID-19 further exacerbated the challenges Syrian refugees faced accessing healthcare in Turkey. Stereotypical representations of Syrian refugees during the pandemic by the local media ignored their specific health challenges [73], some of which included: loss of income as a result of stay-home directives leading to financial constraints and reducing their capabilities to access medical facilities [74]; the difficulties refugees had observing social distancing due to overcrowded and unsanitary urban refugee housing [75]; and the fear of losing their jobs and only source of income meant that many refugees would not report COVID-19 symptoms, thereby increasing the spread within their communities [76].

### 3.3. Policy Recommendations

Overall, Turkey’s healthcare infrastructure is inefficient and inadequate to meet the complex health demands facing Syrian refugees [14]. Conversely to Turkey, Iraq, Jordan and Egypt are primarily facing operational inefficiency rather than scale inefficiency. Therefore, each country faces the unique challenge of providing efficient refugee healthcare services. To address the healthcare needs of Syrian refugees, Turkey needs to increase the number of health personnel, particularly in urban centers, integrate more Syrian health professionals, develop accessible healthcare options for unregistered refugees, regulate and offer support to the informal healthcare sector, and increase financial support to NGOs.

Specifically, strategic scaling of services is required to ensure efficient and effective healthcare provision and protection for Syrian refugees in Turkey, currently hindered by service capacity. Turkey is providing quality services by implementing checks in its procedures. However, research shows that focusing solely on quality depletes performance. Therefore, it is imperative that the system’s capacity and quality are commensurate. A step in the right direction is to continue integrating Syrian health professionals into the Turkish health system. Furthermore, it is important to gradually move away from the temporality of the protection offered. Syrian refugees should be recognized similarly to European refugees under Turkey’s international protection mechanism, necessitating its revision and reassessment. Moreover, a significant portion of Turkey’s over 3.7 million Syrian refugees live in urban and semi-urban cities. Thus, it is necessary as a matter of practicality that integration mechanisms are sought out. Turkey’s economy is in crisis and refugees present a skilled labor force, which could help revitalize the Turkish economy if integrated and given the right opportunities.

More urgently, an amendment in the law allowing refugees access to health services irrespective of the provinces they are registered in is needed. Freedom of mobility is a human right and essential to allowing refugees to lead lives of dignity, seek job opportunities and improve their living conditions, including the ability to access healthcare irrespective of where they find themselves in Turkey.

The process for obtaining temporary protection ID cards is currently bogged down by bureaucracy. It is important that this system is improved, especially since access to healthcare and basic necessities are conditional on these cards. Ideally, persons seeking asylum should be given access to healthcare as soon as they enter Turkey, regardless of their protection status.

Lastly, to increase efficiency, there is a need for effective oversight through an independent governance body capable of pooling the resources of out-care refugee-run clinics, NGOs, INGOs, provincial bodies and state bodies to ensure efficiency in healthcare provision. This process should include decentralizing healthcare administration by shifting some responsibility from the central government to regional or local governments in order to facilitate public healthcare administration for refugees. This model is currently operational in some European countries such as Scotland, Wales and Northern Ireland [77].

## 4. Conclusions

The empirical analysis employed in this study provides practical insights into the performance of refugee healthcare services, which the study found to have relatively declined. Turkey’s inefficient refugee healthcare service is a result of its focus on the quality of service, ignoring the impact of scale. Both factors-operational and scale efficiency must be synergized to fulfil its obligation to provide healthcare to refugees. Even though, Turkey has made great strides to improve protection for Syrian refugees, access to and the quality of healthcare for refugees is underwhelming. While, the quality of trained medical personnel is outstanding, the size of this trained personnel remains inadequate. Efforts to integrate Syrian health professionals has been successful in rural areas, but the same cannot be said of integration in the urban cities. Given that many refugees are moving to urban cities in search of jobs and a better quality of life, the need to ease their access to health care in the urban cities and outside of their provinces of registration remains glaring. Further examination shows that COVID-19 exacerbated the problems Syrian refugees were already facing accessing healthcare in Turkey. As a direction for future research, the amount of medical personnel needed to adequately provide quality services could be estimated. The study could also be extended to the post-COVID-19 restriction period to understand the change in the performance of the healthcare systems.

## Figures and Tables

**Table 1:** Descriptive statistics of efficiency data.

Year	Country	No. of Trained Personnel	Expenditure (Million USD)	No. of Health Consultations
2015	Mean	894	11.38	475,849
	Std. Dev	3284	122.83	1,090,852
	Min.	425	14.03	345,252
	Max.	598	7.20	94,817
2016	Mean	1859	112.63	978,503
	Std. Dev	4038	102.80	962,881
	Min.	285	29.03	371,879
	Max.	740	8.10	98,627
2017	Mean	2980	228.43	1,283,749
	Std. Dev	3290	100.32	1,081,702
	Min.	532	25.32	268,441
	Max.	1213	10.72	119,329
2018	Mean	2776	261.70	1,481,215
	Std. Dev	3983	131.10	942,233
	Min.	637	28.48	263,749
	Max.	1028	15.28	131,285
2019	Mean	3403	345.14	1,816,337
	Std. Dev	4216	133.86	892,693
	Min.	708	33.30	236,581
	Max.	1171	17.97	143,441
2020	Mean	4031	428.58	2,151,459
	Std. Dev	4449	136.61	843,154
	Min.	778	38.11	209,414
	Max.	1315	20.67	155,597

**Table 2:** Efficiency scores descriptive statistics.

Efficiency Dimension		2015	2016	2017	2018	2019	2020
Overall Efficiency	Mean	0.71	0.62	0.56	0.52	0.50	0.49
	Std. Dev	0.27	0.23	0.11	0.11	0.11	0.14
	Min.	0.48	0.45	0.42	0.37	0.35	0.34
	Max.	1.00	1.00	0.71	0.63	0.60	0.67
Pure Technical Efficiency	Mean	0.91	0.88	0.78	0.74	0.71	0.70
	Std. Dev	0.19	0.19	0.19	0.17	0.19	0.22
	Min.	0.57	0.55	0.52	0.59	0.52	0.47
	Max.	1.00	1.00	1.00	0.99	1.00	1.00
Scale Efficiency	Mean	0.80	0.75	0.78	0.78	0.78	0.79
	Std. Dev	0.25	0.20	0.19	0.17	0.17	0.16
	Min.	0.48	0.54	0.59	0.60	0.58	0.58
	Max.	1.00	1.00	0.99	0.99	0.99	0.96

## Data Availability

Publicly available datasets were analyzed in this study. This data can be found here: https://data.unhcr.org/en/situations/syria.
